# Anesthesia Experience With Aortic Valve Replacement Guided by Blood Viscoelasticity Testing in a Late Post-Fontan Patient: A Case Report

**DOI:** 10.7759/cureus.87211

**Published:** 2025-07-03

**Authors:** Tokimitsu Hibino, Yusuke Okui, Yoshie Toba

**Affiliations:** 1 Department of Anaesthesiology, Seirei Hamamatau General Hospital, Hamamatsu, JPN; 2 Department of Anaesthesiology, Seirei Hamamatsu General Hospital, Hamamatsu, JPN

**Keywords:** blood viscoelasticity test, cardiopulmonary bypass (cpb), disorders of fibrinogen, fibrinogen concentrate, fontan-associated liver disease, fontan physiology, surgical aortic valve replacement (savr), thromboelastography (teg)

## Abstract

Coagulation management poses a challenge for post-Fontan patients requiring anesthesia for aortic valve replacement (AVR). This is because post-Fontan patients have a history of multiple sternotomies and resultant strong adhesions, in addition to being potentially deficient in coagulation factors, as observed in several post-Fontan patients. Moreover, coagulation factors are further diluted and consumed during cardiopulmonary bypass (CPB). In this report, we present the anesthetic management during AVR in a post-Fontan patient. A 32-year-old post-Fontan female patient underwent valve replacement for stenosis and regurgitation of the aortic valve. She had a history of multiple thoracotomies and was considered to be at high risk for hemorrhage, based on the assumption that she had extensive and robust adhesions, which were confirmed intraoperatively. Blood viscoelasticity tests were performed preoperatively and after weaning from the CPB to evaluate coagulation factor deficiency. Severe preoperative fibrinogen dysfunction was observed, which was exacerbated after CPB. A marked discrepancy also existed between the results of blood viscoelasticity testing and fibrinogen levels obtained using the Clauss assay. We concluded that the patient exhibited a qualitative functional abnormality of fibrinogen itself, rather than a decrease in fibrinogen concentration. Based on the viscoelasticity tests and clinical hemostatic status, fibrinogen concentrate was administered to treat the hypofibrinogenic state. Evaluation of fibrinogen function using blood viscoelasticity tests was useful in identifying the cause and the treatment of coagulopathy after CPB in our patient.

## Introduction

Recent improvements in diagnostic and treatment modalities have prolonged the life expectancy of post-Fontan patients, exemplified by the rise of the 10-year survival rates to 98% between 2001 and 2012 from 79% between 1973 and 1990, as reported by Pundi et al. [1]. Post-Fontan patients rarely require valve replacement because their heart valves undergo gradual structural changes. In their 47-year experience of treating such patients at a single institution, Graham et al. reported that replacement of the valve in the aortic valve position, i.e., the native aortic valve or Ross procedure-implanted pulmonary artery valve, was performed in 15 of 1,176 post-Fontan patients [2].

Maintaining adequate coagulability during valve replacement in post-Fontan patients is challenging for anesthesiologists. First, to perform surgery, robust and broad adhesions from multiple sternotomies must be dissected. Coagulation factors are consumed during the process of hemostasis from the dissected surface. Second, post-Fontan patients have preexisting deficiencies in coagulation factors [3,4]. Van Nieuwenhuizen reported that the prothrombin time (PT), which reflects the function of coagulation factors II, V, VII, and X, as well as fibrinogen, was prolonged in 79% of post-Fontan patients [3]. Tomita et al. suggested that the congestive state associated with Fontan circulation may cause abnormal liver function and impaired synthesis of proteins, including coagulation factors. However, the causes underlying coagulation factor deficiencies in post-Fontan patients are unclear, and mechanisms such as protein-losing enteropathy (PLE) may have contributory roles [4]. Third, the coagulation factors are further consumed during cardiopulmonary bypass (CPB) [5]. Moreover, coagulation factors decrease owing to blood dilution caused by CPB priming [6].

To the best of our knowledge, few studies have assessed coagulability using thromboelastography (TEG) in open-heart surgery in post-Fontan patients; therefore, herein, we report our experience with anesthetic management in a post-Fontan patient who underwent aortic valve replacement (AVR). The possibility of a hemostatic disorder due to the deficiency of coagulation factors after CPB was posited. Therefore, blood viscoelasticity testing (TEG; TEG®6s, Haemonetics®, Braintree, MA, USA) was used to evaluate the patient’s coagulation function during the perioperative period. We performed TEG three times as follows: before the start of surgery, after weaning from CPB, and after administration of blood transfusion products. Based on the TEG results, fibrinogen concentrate was administered for the hemostatic disorder after weaning from CPB, and hemostasis was achieved.

## Case presentation

A 32-year-old woman (weight: 33.2 kg; body surface area: 1.15 m²) was scheduled to undergo AVR and epicardial conduit upsizing. She was diagnosed with pulmonary atresia and an intact ventricular septum at birth. Since her right ventricle was almost absent, a Fontan-type repair was planned. However, as she had a bicuspid aortic valve and mild stenosis, her physician declined to perform the Fontan procedure. Thus, she did not undergo Fontan surgery and was maintained in Glenn circulation. When the patient was 15 years old, the cyanosis worsened, and she experienced respiratory distress; however, no alternative treatment was available. Therefore, following a detailed preoperative evaluation and catheter coil embolization of the aortopulmonary collateral arteries, she underwent the Fontan procedure. She did well thereafter but developed leg edema at 21 years of age. At age 25 years, she was diagnosed with Fontan-associated liver disease (FALD); at age 29 years, she developed moderate aortic regurgitation; and at age 31 years, she was diagnosed with PLE. The patient also developed infective endocarditis, causing vegetation of the aortic valve, which was treated successfully with antibiotics. However, the maximum pressure gradient of the aortic valve was elevated to 40 mmHg, and aortic valve regurgitation also worsened. Moreover, calcification and stenosis of the epicardial conduit were observed, which contributed to the exacerbation of FALD, PLE, and leg edema. AVR and epicardial conduit upsizing were planned to treat aortic valve and epicardial conduit stenosis, respectively.

**Figure 1:**
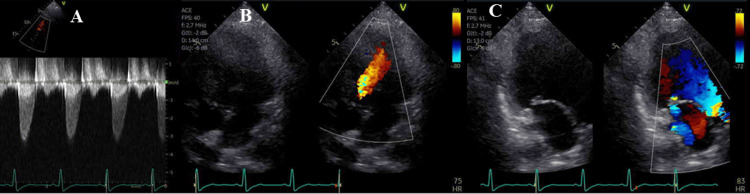
Preoperative transthoracic echocardiography Figure 1A shows the aortic valve velocity. The maximum aortic valve velocity was 3.2 m/s, and the estimated maximum pressure gradient was 41 mmHg. Figure 1B shows aortic regurgitation with the regurgitant jet reaching the apex. Figure 1C shows atrioventricular valve regurgitation. The regurgitant jet is slightly eccentric and directed posteriorly.

Preoperative transthoracic echocardiography revealed a left ventricular ejection fraction of 62%, moderate systemic aortic stenosis (maximum pressure gradient, 40 mmHg), moderate aortic valve regurgitation, and moderate atrioventricular regurgitation (Figure 1). Her liver function was classified as Child-Pugh grade A. Preoperative catheterization revealed a markedly elevated central venous pressure (CVP) of 23 mmHg. Preoperative blood test results are shown in Table 1.

**Table 1:** Preoperative laboratory data

Variable	Our case	Reference range
Blood count		
Hemoglobin (g/dL)	13.8	11.6-14.8
Hematocrit(%)	41.1	35.1-44.4
Platelet count (×10^3^/μL)	103	158-348
Blood chemistry		
Total bilirubin (mg/dL)	0.8	0.4-1.5
Aspartate aminotransferase (U/L)	25	13-30
Alanine aminotransferase (U/L)	18	7-23
Lactate dehydrogenase (U/L)	152	124-222
Creatine phosphokinase (U/L)	22	41-153
Total protein (g/dL)	7	6.6-8.1
Albumin (g/dL)	4.6	4.1-5.1
Blood urea nitrogen (mg/dL)	15	8-20
Creatinine (mg/dL)	0.79	0.46-0.79
Estimated glomerular filtration rate (mL/minute/1.73m^2^)	69	>60
Blood coagulation		
Prothrombin time (s)	13	9.6-13.1
Prothrombin time-international normalized ratio	1.21	0.85-1.10
Activated partial thromboplastin time (s)	42.6	24-34
Fibrinogen (mg/dL)	229	200-400

**Figure 2:**
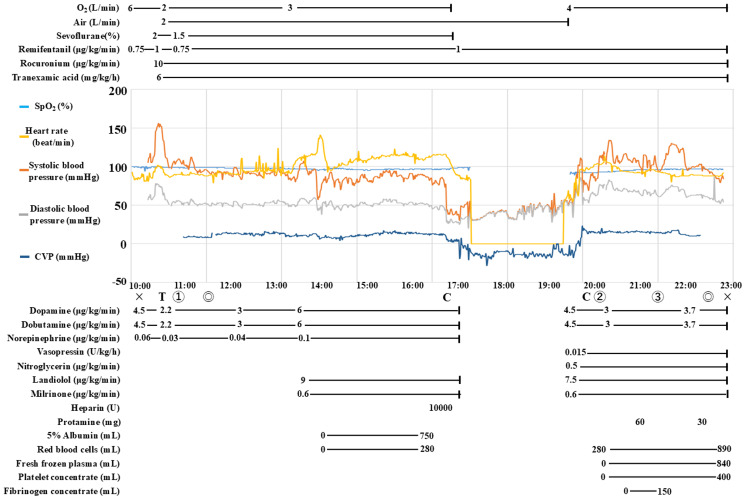
The anesthesia record The “x” represents the start and end of anesthesia, and the duration of anesthesia was 12 hours and 49 minutes. The “◎” indicates the start and end of the surgery, and the duration of the surgery was 11 hours and 1 minute. The “C” represents the start and end of cardiopulmonary bypass, and the cardiopulmonary bypass time was three hours and two minutes. The “T” indicates the timing of intubation. Marks ①, ②, and ③ represent the time that TEG was performed for pre-CPB, post-CPB, and post-transfusion, respectively. The anesthesia record is shown. The vertical axis of the graph represents the values of oxygen saturation (SpO_2_), heart rate, systolic pressure, diastolic pressure, and central venous pressure. Central venous pressure (CVP) is measured as a negative value because vacuum-assisted venous return was used during cardiopulmonary bypass. The horizontal axis of the graph represents time. Because of the wide variety of drugs administered, only heparin and protamine are listed for single-dose drugs, and continuous infusion drugs are listed. Apart from the medications administered as indicated in the anesthesia record, we induced anesthesia with 2.5 mg midazolam, 30 mg ketamine, 30 mg rocuronium, and 4 mg ephedrine. The volume of fluid transfusion was 3870 mL, and the volume of blood transfused was 1170 mL red blood cells, 840 mL fresh frozen plasma, 400 mL platelet concentrates, and 150 mL fibrinogen concentrates. Urine output was 3470 mL and blood loss was 1806 mL.

In the operating room, electrocardiography, arterial blood pressure, CVP, and near-infrared spectroscopy monitoring were performed. The vital signs before anesthesia induction were as follows: blood pressure, 111/75 mmHg; pulse, 86 beats/minute; and oxygen saturation (SpO_2_), 99% (nasal cannula: 2 L/min). Dopamine, dobutamine, and noradrenaline were administered before induction. Anesthesia was induced using midazolam, ketamine, remifentanil, and rocuronium, and maintenance was achieved with sevoflurane, rocuronium, and remifentanil (Figure 2).

**Figure 3:**
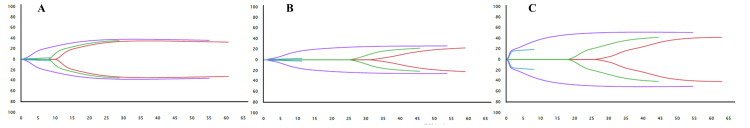
Thromboelastography (TEG) results TEG®6s (Haemonetics®, Braintree, MA, USA) contains four reagents in a cartridge, to each of which a whole blood sample is added, and the time until coagulation occurs and the strength of the blood clot are measured. The purple line indicates CRT and reflects platelet and fibrinogen function. The light blue line indicates CFF and reflects fibrinogen function. The red line shows CK and reflects the function of coagulation factors. The green line indicates CKH, which can antagonize heparin in whole blood samples; by noting the difference between CK and CKH, we can diagnose residual heparin in a patient. The vertical axis of each graph shows the length of the TEG amplitude, in millimeters; the TEG amplitude indicates the clot's intensity. The horizontal axis of each graph is the time from the start of the inspection, in minutes. The reaction time (R) is the time required for the formation of blood clots in each channel and reflects the speed of the coagulation reaction, i.e., the function of the coagulation factors. Figure 3A shows the TEG results for pre-CPB. Since the normal value of the maximum amplitude (MA) of CFF is 15-32 mm, a remarkable fibrinogen dysfunction state can be recognized. Figure 3B shows the results of TEG in post-CPB: compared to pre-CPB (Figure 3A), overall MA is decreased, suggesting reduced clot strength; the Rs of CK and CKH are also prolonged, suggesting decreased coagulation factor function. Figure 3C shows the TEG results for post-transfusion. It is apparent that the overall MA has improved. The recovery of CFF is particularly marked. The Rs of CK and CKH are still prolonged but show improvement from post CPB. CRT: citrated rapid TEG, CFF: citrated functional fibrinogen, CK: citrated kaolin, CKH: kaolin with heparinase, CPB: cardiopulmonary bypass

The first TEG test was performed immediately before surgery. The maximum amplitude (MA) of clot strength measured in the citrated functional fibrinogen assay (CFF-MA) was abnormally low at 2.9 mm (Table 2), suggesting marked impairment of fibrinogen function. Warfarin was discontinued four days prior to surgery and replaced with continuous administration of heparin at 11,200 units/day. The PT/international normalized ratio (PT/INR) during the year prior to surgery was 1.48±0.56. With the discontinuation of warfarin, the PT/INR decreased to 1.03 on the day of surgery. Heparin was discontinued four hours and 30 minutes prior to entering the operating room. Despite a small difference between the kaolin with heparinase assay (CKH-R) and the prolonged clot initiation assay (CK-R), indicating the absence of heparin residuals, the coagulation time was prolonged, with a CK.R time of 10.8 min. The activated clotting time (ACT) measured at this juncture was 134 s. The MA obtained from the citrated rapid test (CRT-MA) was 37 mm (reference range: 52-70 mm) (Figure 3A). The CRT-MA was lower than the normal range, and the platelet count at this time was low at 78000/μL; thus, we suspected a quantitative platelet abnormality rather than a qualitative abnormality. These TEG parameters revealed a deficient state of fibrinogen and coagulation factors, which were consumed during CPB. Therefore, replenishment of fibrinogen and coagulation factors was performed after CPB.

**Table 2:** The thromboelastography (TEG) results in this case and parameters related to hemostasis evaluated simultaneously Results of TEG and blood sampling performed just before surgery, after administration of protamine, and after administration of fibrinogen concentrate and platelet transfusion. The right column shows the normal reference range of each parameter for healthy adults. Post-Fontan patients may have different data distribution. PT: prothrombin time; INR: international normalized ratio; APTT: activated partial thromboplastin time; ACT: activated clotting time; CRT-MA: citrated rapid test-maximum amplitude; CFF-MA: citrated functional fibrinogen assay-maximum amplitude; CK-R: prolonged clot initiation; CKH-R: kaolin with heparinase

Variable	Preoperative	After protamine	After fibrinogen concentrate and platelet transfusion	Reference value
Hemoglobin (g/dL)	12.8	14.8	11.3	12.0-15.0
Platelet (×10^3^/μL)	78	34	71	130-400
Fibrinogen (mg/dL)	229	214	382	200-400
PT (sec)	11.1	14.9	13.9	11-13.2
PT/INR	1.03	1.4	1.3	0.8-1.2
APTT (sec)	35.6	36.6	34.4	25-41
ACT (sec)	134	117	106	70-140
CRT-MA (mm)	37	23.4	49.6	52-70
CFF-MA (mm)	2.9	2.5	18.7	15-32
CK-R (min)	10.8	33.7	28	4.6-9.1
CKH-R (min)	8.7	26.7	19.1	4.3-8.3

Prior to CPB initiation, the surgeons required five hours for careful dissection of the strong adhesions. Following dissection, 10000 IU of heparin was administered, achieving an ACT of 999 seconds. The aortic valve was replaced, and the epicardial conduit was upsized. The patient’s body temperature was maintained at 35.2°C during CPB. She was smoothly weaned off CPB. After modified ultrafiltration, which was implemented for the purpose of infusion volume management and removal of inflammatory mediators, the aortic and venous cannulas were removed, and 140 mg of protamine was administered.

A second TEG was performed after the completion of protamine administration. The CFF-MA was only 2.5 mm, which was even lower than the preoperative value, suggesting severe impairment in fibrinogen function. The CK-R increased to 33.7 minutes, which was greater than the preoperative value, suggesting impaired coagulation factor function. The reaction time measured in the CKH-R was 26.7 minutes, which was seven minutes lower than the CK-R. Furthermore, the CRT-MA was 23.4 mm, indicating that the clot strength was even lower than the preoperative value, suggesting further quantitative impairment of platelet function as well as possible qualitative impairment due to CPB (Figure 3B). In contrast to these abnormal TEG parameters, the simultaneously measured ACT was 117 seconds, apparently within the normal range.

Based on the second TEG result, we transfused 3 g of fibrinogen concentrate and 20 units of platelets. After the blood products were administered, a third TEG was performed. The ACT recorded at this time was 107 seconds, which did not differ greatly from the value at the preceding measurement. However, the CFF-MA levels increased to within the normal range at 18.7 mm. The CRT-MA was 49.6 mm, representing a marked improvement over the value recorded at the second TEG. Despite improvements from the second TEG, a CK-R of 28 minutes and CKH-R of 19.1 minutes indicated that coagulation factor function was still impaired. The difference between CK-R and CKH-R was 8.9 minutes, which was greater than that for the second TEG. We suspected the presence of residual heparin and administered an additional 30 mg of protamine (Figure 3C).

Surgery was completed after careful hemostatic treatment. Red blood cells and fresh-frozen plasma were transfused at 1170 mL and 840 mL, respectively. The postoperative course was good; the patient left the intensive care unit on postoperative day 8 and was discharged on postoperative day 23.

## Discussion

Deficiencies in coagulation factors V, VII [3], and XIII [4], resulting in a prolonged PT, have been reported in post-Fontan patients. In the present case, the fibrinogen level was within the normal range (229 mg/dL), and the PT was not prolonged; however, the results of TEG were contradictory.

The CFF-MA was only 2.9 mm (normal range: 15-32 mm), suggesting a marked decrease in fibrinogen function (Figure 3-A). Demailly et al. examined the correlation between the MA-functional fibrinogen maximum amplitude (MA-FFMA) measured using TEG6S and fibrinogen concentration (Clauss assay) and reported a correlation coefficient of 0.79 (0.55-0.91), concluding that a correlation existed between TEG measurements and fibrinogen concentration [7]. However, in our case, the CFF-MA values and fibrinogen concentration were widely discrepant. If we had assessed fibrinogen function based solely on fibrinogen values derived from the Clauss assay, we might have overestimated the fibrinogen function in our patient. Moreover, the CK-R was 10.8 min, exceeding the normal range of 4.6-9.1 minutes, suggesting decreased coagulation factor function.

The CRT-MA was 37 mm, which also fell below the normal range of 52-70 mm. Although CRT-MA is reportedly correlated with platelet function [8], in retrospect, we were skeptical about platelet dysfunction for two reasons. First, the preoperative platelet level was only 78000/μL, and the low platelet count could have been responsible for the low CRT-MA value. Second, CRT-MA reflects both platelet and fibrinogen function, and a marked decrease in fibrinogen function may have contributed to the low CRT-MA value.

In this case, we performed the second TEG after protamine administration. The ACT (117 seconds) and fibrinogen level (214 mg/dL) measured at the same time point were within the normal range. The platelet count was 34000/µL. No clot formation occurred in the operative field, and coagulopathy was clinically evident. Judging from the ACT, fibrinogen level, and platelet count, the main cause of the coagulopathy at this juncture was considered to be a low platelet count, which could have been primarily treated with platelet transfusion. However, TEG revealed CFF-MA and CRT-MA values of 2.5 mm and 23.4 mm, respectively (Figure 3B). Platelet function seemed to be impaired by CPB, but the decline in fibrinogen function was more severe. We administered 3 g of fibrinogen concentrate and 20 units of platelets. The third TEG indicated that these blood product administrations were adequate.

Congenital fibrinogen disorders are classified into two main categories, viz., quantitative (type 1) and qualitative fibrinogen abnormalities (type 2) [9]. Qualitative abnormalities of fibrinogen are characterized by normal or reduced fibrinogen levels, albeit with a disproportionate decrease in fibrinogen function [9]. Acquired fibrinogen dysfunction may be observed in patients with liver diseases, including alcoholic cirrhosis, post-necrotic cirrhosis, chronic active liver disease, and severe hepatitis. Martinez et al. reported that liver disease-associated acquired fibrinogen dysfunction is a disorder of fibrin monomer polymerization and a qualitative fibrinogen abnormality [10]. Fibrinogen from patients with liver disease was found to have higher sialic acid content. Interestingly, the function of fibrinogen was normalized by desialylation of this fibrinogen [10]. Martinez et al. observed structural changes in fibrinogen in patients with severe liver disease [10], whereas our patient exhibited relatively preserved liver function, as evidenced by the Child-Pugh class A classification.

However, a different hypothesis emerges when this case is observed from the perspective of CVP. According to Noguchi's study that summarized the course of Denver peritoneovenous shunt creation for patients with cirrhosis, the median (minimum-maximum) CVP of 24 patients with decompensated cirrhosis was 10 (4-19) mmHg and did not exceed 20 mmHg in any patient [11]. The preoperative CVP in our patient was 23 mmHg, which was higher than that of the group of patients with decompensated cirrhosis. Although our patient’s liver function was scored as Child-Pugh class A, her CVP might have been higher than that of the patients reported by Martinez et al. It is possible that structural changes in fibrinogen could have occurred in our patient, as in the cases reported by Martinez et al.

To our knowledge, there are no reports on the sialic acid content of fibrinogen in patients with FALD. It is unknown whether the sialic acid content is higher in the fibrinogen of patients with FALD. Therefore, the cause of the discrepancy between fibrinogen levels and CFF-MA observed in our patient remains unclear. The following possible causes of the discrepancy between fibrinogen levels and CFF-MA can be posited. The first is a phenomenon specific to our patient, and the second is a coagulation factor deficiency specific to the Fontan circulation [3,4]. The third, which is merely our assumption, is that the previously mentioned structural qualitative abnormalities in fibrinogen are specific to patients with FALD. In order to understand the cause of the abnormal fibrinogen function in patients with FALD, the structural changes in fibrinogen in these patients should be investigated. Studies should also be conducted to evaluate the correlation between TEG data, other coagulation parameters, and intraoperative clinical hemostatic status in patients with FALD. We believe that it is important to use TEG to assess the actual function of fibrinogen in post-Fontan patients.

## Conclusions

Few studies have reported on the use of TEG to evaluate coagulability during valve replacement in post-Fontan patients. In this study, we report our experience with anesthetic management for AVR in a patient with FALD using TEG. TEG showed a discrepancy between the CFF-MA and fibrinogen levels, proving useful in identifying and treating the cause of coagulopathy after CPB weaning. We believe that qualitative fibrinogen abnormalities might exist in patients with FALD, and the actual functional impairment may be disproportionate to the normal fibrinogen levels. However, this is a hypothesis derived from only a single case report, and no conclusion can be drawn from the present findings. Therefore, future studies conducting an in-depth evaluation of fibrinogen levels and CFF-MA in patients with FALD are warranted. Similarly, multicenter studies should be conducted to ascertain the correlation between TEG results and both other coagulation parameters and the intraoperative clinical hemostatic status.
